# Anticonvulsant Effect of *Cicer arietinum* Seed in Animal Models of Epilepsy: Introduction of an Active Molecule with Novel Chemical Structure

**DOI:** 10.6091/ibj.1391.2014

**Published:** 2015-01

**Authors:** Soroush Sardari, Motahareh Amiri, Hourieh Rahimi, Mohammad Kamalinejad, Jamshid Narenjkar, Mohammad Sayyah

**Affiliations:** 1 *Biotechnology Research Center, Drug Design and Bioinformatics Group, Pasteur Institute of Iran, Tehran, Iran; *; 2 *Dept. of Physiology and Pharmacology, Pasteur Institute of Iran, Tehran, Iran;*; 3 *Dept. of Pharmacology, Faculty of Medicine, Shahed University, Tehran, Iran;, *; 4 *Dept. of Pharmacognosy, Faculty of Pharmacy, Shaheed Beheshti University of Medical Sciences, Tehran, Iran*

**Keywords:** Anticonvulsants, *Cicer*, Kindling, Pentylenetetrazole (PTZ)

## Abstract

**Background:** *Cicer arietinum* (Chickpea) is one of the most important harvests in the world with high nutritional value. Lack of essential oils in the seeds of Chickpea is an advantage in search for drug-like molecules with less toxicity. We evaluated anticonvulsant effect of *C. arietinum *in common animal models of epilepsy. **Methods:** Dichloromethane extract was obtained from *C. arietinum* seeds by percolation. Acute toxicity of the extract was assessed in mice. Protective effect of the extract was examined against tonic seizures induced by maximal electroshock (MES; 50 mA, 50 Hz, 1 s) in mice, clonic seizures induced by pentylenetetrazole (PTZ; 60 mg/kg, i.p.) in mice, and electrical kindling model of complex partial seizures in rats. The extract was fractionated by n-hexane to *f1* and *f2* fractions. The extract and fractions underwent phytochemical analysis by thin layer chromatography. The active anticonvulsant fraction, *f1,* was subjected to matrix assisted laser desorption/ionization (MALDI) mass analysis. **Results:** The crude extract had neither toxicity up to 7 g/kg nor protective activity in MES and kindling models. However, it significantly inhibited clonic seizures induced by PTZ. *f1* fraction mimicked protective effect of the extract. Phytochemical screening revealed the presence of considerable amount of alkaloids in the extract and fractions. Moreover, a novel structural class was detected in *f*1 fraction. **Conclusion:** Finding an anticonvulsant molecule pertaining to a new structural class in the seeds of *C. arietinum* promises an effective and inexpensive source of antiepileptic medication. Further studies are needed to identify its mechanism of action and more clues into its structure-activity relationship.

## INTRODUCTION

Epilepsy is the third most common neurological disorder after stroke and Alzheimer's disease [1]. Treatment of epilepsy was improved by several third generation of antiepileptic drugs during the past three decades. Nevertheless, resistance to antiepileptic drugs as well as intolerability in 20-30% of the patients led to serious demands for developing new drugs or strategies for epilepsy treatment [2]. One of the approaches to search for new antiepileptic drugs is the investigation of naturally-occurring compounds, which may belong to new structural classes.

Chickpea (*Cicer arietinum* L., Fabaceae) is the third most important pulse crop (after beans and peas) in the world, and it has been used for its nutritional properties since 7,000 BC [3]. Similar to most other plants of Fabaceae family, Chickpea has no or negligible amount of essential oils. Essential oils often have high potential toxicity and narrow therapeutic index [4]. Furthermore, their particular chemical structure has weak potential for modification. Therefore, they are not superior candidate for drug design [5, 6]. Thus, lack of essential oils can be considered as an advantage and favored Chickpea in search for safe and effective medicines pertaining to new structural classes. We have previously reported the anticonvulsant activity of some plants from Fabaceae family [7-9]. In this study, anticonvulsant and toxic effects of *C. arietinum*, the most famous plant of this family, was evaluated in several experimental models of epilepsy, including pentylenetetrazole (PTZ) model of clonic seizures, maximal electroshock (MES) model of tonic seizures, and amygdala kindling, which is the model of complex partial seizures with secondarily generalization. 

## MATERIALS AND METHODS

***Plant materials.*** *C. arietinum *was purchased from a local market. The plant was authenticated, and the voucher specimen (No. 8104) was deposited in the Herbarium of Pasteur Institute of Iran, Tehran.

***Chemicals. ***PTZ, phenytoin sodium, ethosuximide, and lamotrigine were purchased from Sigma-Aldrich (Germany). Ketamine was obtained from Rotex Medica (Germany) and xylazine from Candelle (Ireland). Tween 80, polyethylene glycol, methanol, ethyl acetate, toluene, DMSO, ethanol, dichloromethane, n-hexane, Dragendorrf's reagent, potassium hydroxide, glacial acetic acid, vanillin, sulfuric acid, hydrochloric acid, and sodium hydroxide were purchased from Merck (Germany). PTZ, phenytoin sodium, lamotrigine, and ethosuximide were dissolved in normal saline (0.9% w/v). The extract and its fractions were dissolved in Tween 80 (25%): DMSO (3:1 v/v). All solutions were prepared freshly. 

***Extract preparation.*** *C. arietinum* seed (50 g) was powdered and extracted by percolation method using dichloromethane (150 ml) at the room temperature for 48 h. The extract was then concentrated by a rotary evaporator at temperature not exceeding 50°C. The yield of the extract was 30% (w/w). The extract was stored at 4°C throughout the experiments. 

***Fractionation. ***Five ml of n-hexane was added to 5 g of the extract. The mixture was vortex mixed and centrifuged at 129 ×g for 10 min. The supernatant (yellowish color) was separated, and n-hexane (5 ml) was again added to the pellet, vortex mixed and centrifuged at 129 ×g for 10 min. The supernatant was then separated. The procedure was repeated three times until a colorless supernatant (organic phase) was obtained. The collected organic phase was concentrated by a rotary evaporator. Finally, two fractions were obtained, a white precipitate (1.5 g) as *f1*, and a concentrated yellowish organic phase (2.3 g) as *f2*. 

***Preliminary phytochemical screening.*** The crude extract of *C. arietinum *and the fractions were screened for the presence of saponins, alkaloids, flavonoids, anthraquinones, anthrones, coumarins, valepotriates, and essential oil by thin layer chromatography using silica gel G (Merck) plates of 0.25 mm thickness [10]. The extract was dissolved in Tween 80 (25%): DMSO (3:1 v/v). Development of thin layer chromatography plates was carried out by ethyl acetate:methanol:water (100:13.5:10 v/v/v) and ethyl acetate:toluene (93:7). Then, the plates were sprayed with the following reagents to visualize the spots and detect respective classes of compounds: Dragendorrf's reagent (alkaloids), potassium hydroxide (anthraquinones, anthrones, and coumarins), hydrochloric acid and glacial acetic acid mixture (valepotriates), vanillin and sulfuric acid mixture (essential oil), and polyethylene glycol (flavonoids). Reagents were prepared according to Stahl method [11], and detection was carried out visually in visible light and under UV light (λ = 365 nm).

***Spectroscopic analysis. ***matrix assisted laser desorption/ionization (MALDI) mass analysis was performed for *f1* fraction with Bruker 9.4T Apex-Qe FTICR instrument and the Data Analysis 3.4. Nuclear magnetic resonance (NMR) spectroscopy was carried out on VNMR 500 instrument, and sample was dissolved in d6-DMSO.

***Animals. ***Male NMRI mice (20-28 g) and male Wistar rats (270-320 g), which was obtained from Pasteur Institute of Iran (Tehran), were used throughout the study. The animals were housed in standard cages (10 mice or 4 rats in each cage) with free access to food (standard laboratory rodent's chow) and water. The animal room temperature was maintained at 23 ± 1°C with a 12-h light/12-h dark cycle (light on from 06:00 a.m.). The study was approved by the Ethics Committee of Pasteur Institute of Iran and conformed to the European Communities Council Directive of 24 November 1986 (86/609/EEC). All animal experiments were carried out in such a way to minimize the number of animals and their suffering. Each animal was tested once, and all injections were carried out i.p. in a volume of 0.1 ml/10 g of mice and 1 ml/kg of rat body weight.

***Acute toxicity.*** Different groups of mice (10 mice in each group) were treated i.p. with the solvent of the extract and fractions (10 ml/kg, as control) as well as different doses of the extract (4, 5, and 7g/kg) and the fractions (2 and 4g/kg). The mortality rate was recorded 24 h thereafter.

***Maximal electroshock-induced seizures. ***Electro-convulsive shock inducing Hind Limb Tonic Extension in 99% of the animals was previously determined [9]. The electrical stimulus (50 mA, 50 Hz, 1-s duration) was applied through ear clip electrodes using a stimulator apparatus. Six groups of 10 mice each pretreated i.p. with the extract (2, 4, and 5 g/kg), the solvent of the extract (10 ml/kg, as control), phenytoin (25 mg/kg, as positive control), and saline (10 ml/kg, as control). After 30 min, the animals received transauricular electroshock. Abolition of Hind Limb Tonic Extension within 10 s after delivery of the electroshock was the criterion for anticonvulsant effect.

***PTZ-induced seizures.*** The minimal i.p. dose of PTZ at which 99% of the animals showed general clonus was determined by a dose-percent effect curve as previously described [7]. General clonus was considered as the criterion of clonic seizure, which characterized by clonus of four limbs with transient loss of righting reflex [7]. The extract (2, 4, and 5 g/kg) and fractions (2 and 4 g/kg), the solvent of the extracts and the fractions (10 ml/kg, as control), ethosuximide (150 mg/kg, as positive control), and saline (10 ml/kg, as control) were injected to the mice (10 different groups, 10 animals in each group). After 30 min, PTZ (60 mg/kg) was injected into the animals. If no general clonus occurred during a 30-min period of observation, the animals were considered protected. 

***Stereotaxic surgery and kindling procedure. ***Rats were anesthetized with i.p. injection of 60 mg/kg ketamine and 10 mg/kg xylazine. The animals were then stereotaxically implanted with bipolar stimulating and monopolar recording stainless-steel Teflon-coated electrodes (A-M Systems, USA, twisted into a tripolar configuration) in the basolateral amygdala (coordinates: anterior, -2.5 mm from bregma; lateral, 4.8 mm from bregma, and ventral, 7.3 mm from dura) of the right hemisphere [12]. Another electrode was connected to a skull screw, placed above the left cortical surface, as earth and differential electrode. The electrodes were fixed to the skull with dental acrylic. The animals were given seven days for recovery after surgery, before the kindling protocol to be started. One week after surgery, after discharge (AD) threshold was determined in amygdala by a 2-s stimulus (frequency: 100 Hz, shape: monophasic square wave of 1 ms per pulse). The stimulation was initially delivered at 50 µA and then at 5-min intervals. Increasing stimulus intensity in increments of 50 µA was delivered until at least 5 s of AD was recorded [13]. Then, the animals were stimulated at AD threshold once daily until three consecutive stage five seizures were elicited according to Racine classification as stage 1, facial clonus; stage 2, head nodding; stage 3, forelimb clonus; stage 4, rearing and bilateral forelimb clonus; stage 5, rearing, loss of balance and falling [14]. These animals were then considered fully kindled. The extract (5 g/kg), solvent of the extract (1 ml/kg, as control), lamotrigine (100 mg/kg, as positive control), and saline (10 ml/kg, as control) were injected into the kindled rats (four different groups, six rats in each group). After 30 min, amygdala was stimulated at AD threshold. AD duration, behavioral seizure severity, and duration of stage five seizure behavior was then measured in each animal.

***Data analysis. ***Statistical analysis was performed by SPSS for windows software version 16.0. The number of mice showing tonic or clonic seizures in MES and PTZ tests as well as the number of rats showing stage five seizure behaviors were expressed as the percentage of the animals with convulsions. Fisher's exact test was also used to analyze the data. The dose of the extract produced anticonvulsant effect in 50% of animals (ED_50_) was calculated by log-probit analysis. The latency time to occurrence of clonic seizures, AD duration, and S5 were expressed as mean ± S.E.M. and analyzed by one-way analysis of variance (ANOVA) and Tukey's post test. * P* value of less than 0.05 was the critical criterion for statistical significance.

## RESULTS

***Mortality. ***Neither any mortality nor abnormality in mice behavior was observed after administration of the extract (up to dose of 7 g/kg) and fractions (up to dose of 4 g/kg).

***Anticonvulsant activity.*** Maximum non-toxic doses of the extract and fractions had no anticonvulsant activity against seizures induced by MES (Table 1). However, clonic seizures induced by PTZ were inhibited by the extract, and ED_50_ value of 3.4 g/kg was obtained for the extract (Table 2). After fractionation of the extract, significant anticonvulsant activity in PTZ test was observed in non-organic phase (*f1* fraction) (Table 2). The extract had no inhibitory effect on kindled seizures (Table 3). 

**Table 1:** Effect of *C. arietinum *extract on tonic seizures induced by maximal electroshock in mice

**Treatment**	**Dose**	**Incidence of tonic seizures (%)**
Control 1	10 ml/kg	100
Control 2	10 ml/kg	90
Phenytoin	25 mg/kg	0***
*C. arietinum* extract	5 g/kg	100

n = 10,

٭٭٭ *P*<0.001 compared to control value. Control 1, saline, solvent of phenytoin; control 2: Tween 80 (25%):DMSO (3:1, v/v), solvent of the extract and fractions.

**Table 2:** Effect of *C. arietinum *extract and fractions on clonic seizures induced by pentylenetetrazole in mice

**Treatment**	**Dose**	**Incidence of clonic seizures (%)**	**Latency of clonic seizures (s)**
Control 1	10 ml/kg	100	137.9 ± 11.3
Control 2	10 ml/kg	100	130.5 ± 11.3
Ethosuximide	150 mg/kg	0***	-
*C. arietinum *extract *C. arietinum *extract *C. arietinum *extract *f1* fraction *f1 *fraction *f*2 fraction *f*2 fraction	2 g/kg 4 g/kg 5 g/kg 2 g/kg 4 g/kg 2 g/kg 4 g/kg	70 40* 40* 60 40* 80 90	219.14 ± 56.37 291 ± 54.87 630 ± 166.5*** 304.3 ± 125.3* 624.3 ± 111.4*** 291.5±47.25 242.33±21.48

n = 10,

٭ *P*<0.05

٭٭٭ *P*<0.001 compared to control value. Control 1: Saline, solvent of ethosuximide; control 2:Tween 80 (25%):DMSO (3:1, v/v), solvent of the extracts and fractions.

***Preliminary phytochemical analysis. ***The extract consisted of alkaloids and valepotriates, while both fractions were only shown to have alkaloids (Table 4). In addition, saponins, flavonoids, anthraquinones, anthrones, coumarins, and essential oil were absent in the extract.

**Fig. 1:**
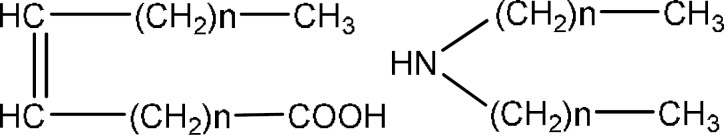
Suggested chemical structure of the compound present in *f1* fraction of *C. arietinum* seed extract.

***Spectroscopic analysis. ***MALDI mass results exhibited the following peaks: 689.21068, 637.32377, 541.17371, 527.15799, 489.31045, 449.36009 (possible solvent impurity/stabilizer), 413.26614, and 393.29762 (possible solvent impurity/stabilizer). Normal impurities are dioctyl-adipate and dioctyl sebacate. The NMR was carried out in DMSO on a cold probe on the departments 500 MHz NMR. Due to the low solubility, the signal was collected on the cold probe overnight, equivalent to collecting data for 18 days on a standard probe. The peaks are as follows: 0.8 (~6H), 1.3 (~20H), 1.5 (~2.5H), 2.0 (~3H), 5.3 (~3H), 2.3 (~2H), 2.7 (~1.1H), and 8.5 (~1H). Chemical and spectroscopic results demonstrated that a compound with structural type shown in Figure 1 exists in the *f1* fraction. 

## DISCUSSION

Fabaceae, which is the third largest family of flowering plants, has been widely studied in search for safe and effective antiepileptic medicines [7-9].

PTZ and MES are the most commonly used preliminary screening tests for characterizing potential anticonvulsant drugs. The PTZ test represents a valid model for human generalized myoclonic and also absence seizures while MES test is considered to be a predictor of likely therapeutic efficacy against generalized tonic-clinic seizures [15]. 

The present study is the first report demonstrating that *C. arietinum* seeds possess anticonvulsant activity against clonic seizures induced by PTZ with ED_50_ value of 3.4 g/kg. Although the extract had low potency, it showed high safety. Therefore, further activity-guided fractionation and phytochemical analysis was performed to clarify the nature of the anticonvulsant compound(s) present in the extract. We found that *f1* fraction of the extract had the same anticonvulsant potency as the crude extract. The phytochemical analysis revealed that alkaloids are the main constituent of *f1*. The anticonvulsant activity of alkaloids has been proven previously [16]. Therefore, the anticonvulsant activity of the extract and *f1* fraction could be attributed to the activity of alkaloids present in the plant.

**Table 4:** Components of the crude extract and fractions of the seeds of *C. arietinum*

***Compound class***	***Dichloromethane extract***	***f1*** *** fraction***	***f2 *** ***fraction***
Saponins	-	-	-
Alkaloids	+	+++	++
Flavonoids	-	-	-
Antraquinons/antrones/coumarins	-	-	-
Essential oils	-	-	-
Valepotriates	+	-	-

+: positive; -: negative.

**Table 3:** Effect of *C. arietinum *extract on kindled seizures in rats

**Treatment**	**Dose**	**Incidence of S5 (%)**	**ADD (s)**	**S5D (s)**
Control 1	10 ml/kg	100	78.9 ± 4.4	18.6 ± 1.6
Control 2	10 ml/kg	90	73.3 ± 4.7	19.4 ± 2.5
Lamotrigine	20 mg/kg	0***	35.7 ± 2.4***	0 ± 0***
*C. arietinum* extract	5 g/kg	100	75.4 ± 4.9	16.9 ± 6.8

n = 6,

٭٭٭ *P*<0.001 compared to control value. Control 1, saline, solvent of Lamotrigine; control 2, Tween 80 (25%):DMSO (3:1, v/v), solvent of the extract; S5: generalized seizures; ADD, after discharge duration; S5D: duration of generalized seizures

**Fig. 2:**
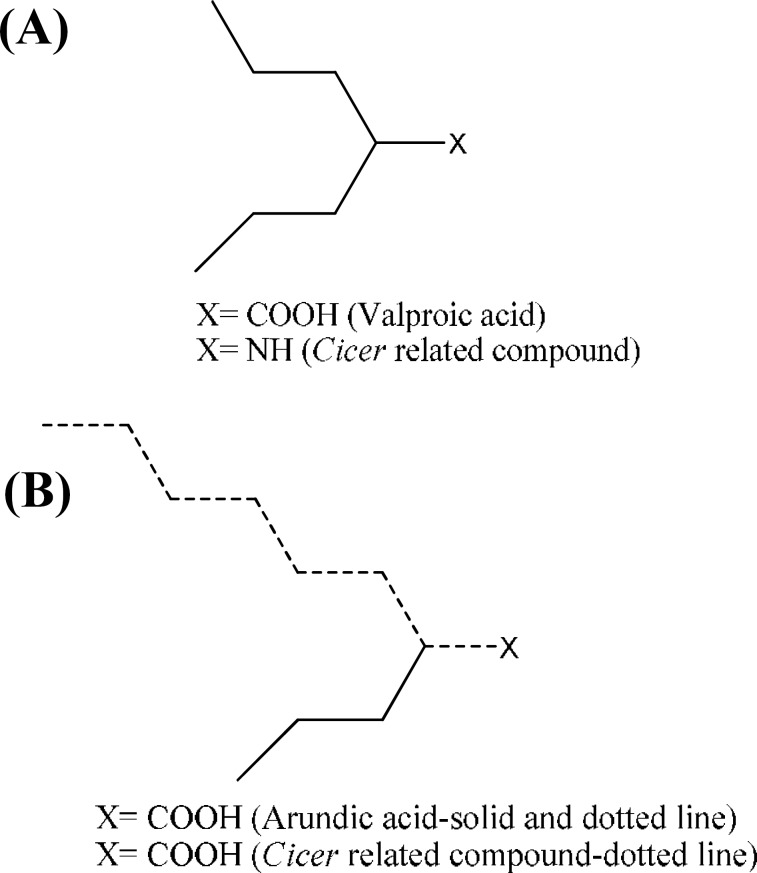
The molecular fragment similarities between *Cicer*-related compound and the anticonvulsant drugs valproic acid **(A)**, and arundic acid **(B)**.

**Fig. 3:**
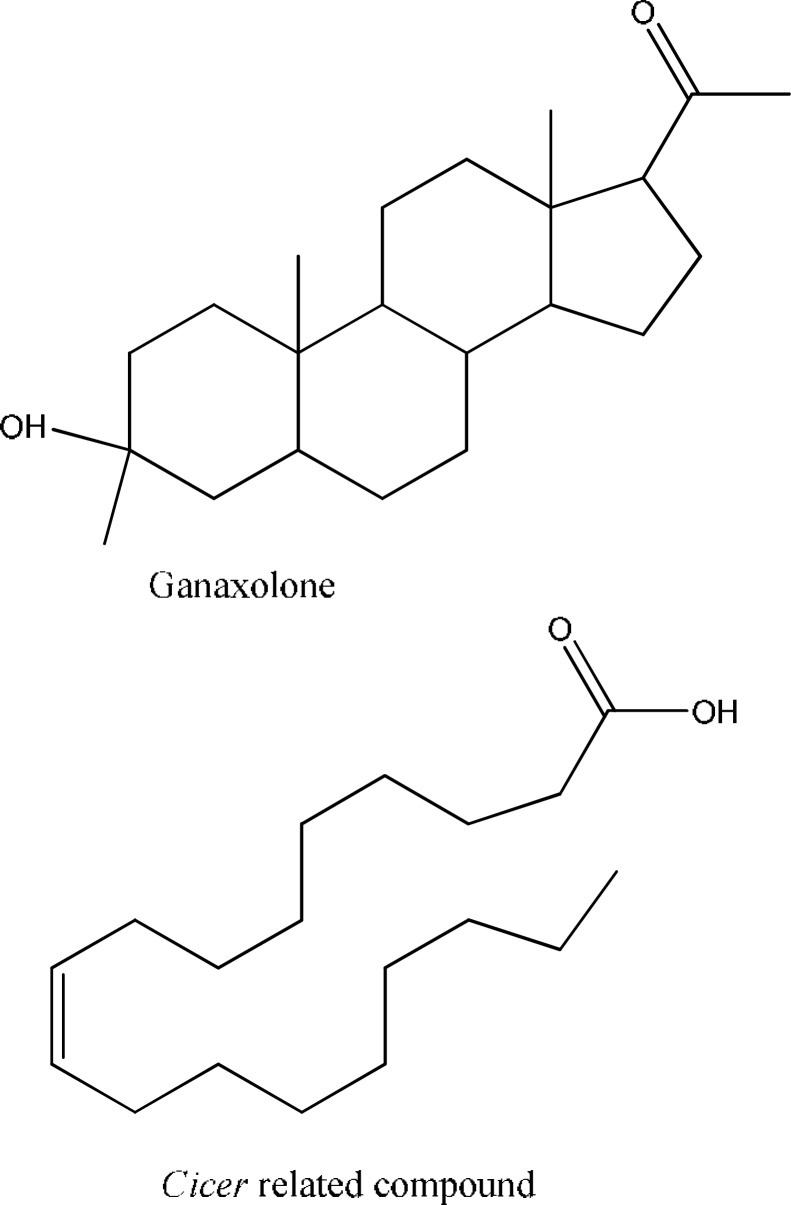
Chemical sructure of Cicer-related compound and ganaxolone. Note the similar scaffold of the compounds in terms of carbon numbers tracing the chain length and overall shape after folding.

MALDI mass analysis performed on *f1* identified a compound with a new structural class as a combination of two moieties possibly connected through electrostatic or hydrogen bonds. The compound contained two hydrocarbons with COOH and NH functional groups attached to it (Fig. 1). In addition to presence of the functional groups necessary for anticonvulsant activity, *Cicer*-ralated compound has fragmental similarities with the standard antiepileptic drug valproic acid and its derivative, arundic acid [17], which are demonstrated in Figure 2. Furthermore, there is another synthetic derivative of neurosteroid allopregnanolone, called ganaxolone, which is a powerful modulator of GABA_A_ receptors [18]. Ganaxolone has protective activity in rodent seizure models, including clonic seizures induced by PTZ. As shown in Figure 3, *C. arietinum*-related compound has strong similarity to the main scaffold of ganaxolone. 

The evidence in similarity could further indicate correlation of the isolated compound to the present bioactive anti-convulsants. Furthermore, it supports our future investigation to identify its mechanism and sheds light on its structure-activity relationship.

The dichloromethane extract of *C. arietinum *and its *f1* fraction possess protective effect against PTZ-induced clonic seizures. Finding an anticonvulsant molecule pertaining to a new structural class in the seeds of *C. arietinum* with acceptable potency and lack of toxicity promises an effective and inexpensive source of antiepileptic medication. Further studies are needed to identify its mechanism of action and more clues into its structure-activity relationship.
